# MRI in Febrile Children: Temperature Changes in Sedated or Anesthetized Pediatric Patients With Initial Body Temperature ≥ 38.5°C

**DOI:** 10.1002/pan.70187

**Published:** 2026-04-11

**Authors:** Hendryk Schneider, Daniel Matheisl, Kristin Goller‐Bruchmann, Martin Kuntz, Hans Fuchs

**Affiliations:** ^1^ Center for Pediatrics, Division of Neonatology and Pediatric Intensive Care Medicine, Medical Center‐University of Freiburg, Faculty of Medicine University of Freiburg Freiburg Germany; ^2^ Department of Diagnostic and Interventional Radiology, Division of Pediatric Radiology, Medical Center‐University of Freiburg Faculty of Medicine, University of Freiburg Freiburg Germany

**Keywords:** children, fever, hypothermia, MRI, sedation

1

Magnetic resonance imaging (MRI) is an indispensable diagnostic modality in pediatric medicine, providing high‐resolution imaging without ionizing radiation [1]. Because young children are often unable to remain motionless, sedation or general anesthesia is frequently required to ensure image quality [2]. Both MRI and anesthesia influence thermoregulation, creating concern regarding temperature instability during imaging.

Thermal balance during pediatric MRI reflects two opposing mechanisms. Radiofrequency (RF) electromagnetic fields deposit energy in tissue, quantified as specific absorption rate (SAR), which may lead to temperature elevation under certain conditions [3, 4]. Conversely, sedation and general anesthesia impair thermoregulatory control through redistribution of core heat to the periphery, reduced metabolic heat production, and suppression of vasoconstriction and shivering. Combined with the cool ambient environment of MRI suites and limited access for active warming, anesthetized children are particularly vulnerable to hypothermia [5, 6, 7].

Although several pediatric studies have demonstrated net cooling during MRI, febrile children have systematically been excluded, including in the recent observational cohort by Madsen et al. [5]. As a result, no empirical data exist describing temperature changes in children undergoing MRI with elevated pre‐scan body temperature. This represents a clinically important gap, as urgent MRI is often required in febrile children with suspected central nervous system or deep neck infections, as well as febrile status epilepticus.

We therefore retrospectively analyzed all pediatric intensive care and emergency department patients at our tertiary center who underwent emergency MRI between January 2018 and December 2023 with a documented pre‐scan tympanic temperature ≥ 38.5°C. All examinations were performed on a 1.5‐Tesla scanner. Pre‐scan (T1) and post‐scan (T2) temperatures were compared using the Wilcoxon matched‐pairs signed‐rank test. Institutional review board approval was obtained.

Sixteen children met inclusion criteria. Patient characteristics (including MRI indications and body temperature changes) are summarized in Table 1.

**TABLE 1:** Patient characteristics including temperature changes, sedation/anesthesia medication, and MRI parameters (*n* = 16).

Characteristic	Value
Age, years, median (IQR)	2.3 (1.1–7.5)
Weight, kg, median (IQR)	15.5 (10.5–23.5)
Time in MRI suite, min, median (IQR)	60 (45–60)
Time from temperature measurement (T1) to MRI start, min, median (IQR)	17.5 (10–30)
Time from MRI end to temperature measurement (T2), min, median (IQR)	15 (6.3–51.3)
Pre‐scan temperature, °C, median (IQR)	38.7 (38.5–39.1)
Post‐scan temperature, °C, median (IQR)	37.8 (37.1–38.3)
Paired temperature difference, °C, median (IQR)	−1.15 (−1.475 to −0.775)
Sedation (p.o. promethazine, p.o. chloral hydrate, i.v. phenobarbital or i.v. propofol), *n* (%)	6 (37.5%)
General anesthesia (continuous infusion of midazolam or propofol), *n* (%)	10 (62.5%)
Antipyretics prior to MRI, *n* (%)	9 (56%)
*MRI modality*	
Brain/cranial MRI, *n* (%)	10 (63%)
Neck MRI, *n* (%)	4 (25%)
Thoracic MRI, *n* (%)	1 (6%)
Abdominal MRI, *n* (%)	1 (6%)
*Indication for MRI*	
Deep neck infection, *n* (%)	5 (31%)
Status epilepticus, *n* (%)	3 (19%)
Mastoiditis/sinusitis, *n* (%)	3 (19%)
Acute impaired consciousness, *n* (%)	3 (19%)
Urosepsis with renal abscess, *n* (%)	1 (6%)
Ventriculo‐peritoneal shunt infection, *n* (%)	1 (6%)

*Note:* Values are presented as median (interquartile range) or number (percentage).

Abbreviations: IQR, interquartile range; MRI, magnetic resonance imaging; T1, pre‐scan temperature measurement; T2, post‐scan temperature measurement.

Median pre‐scan temperature was 38.7°C (IQR 38.5–39.1), decreasing to 37.8°C post‐scan (IQR 37.1–38.3). All patients demonstrated a reduction in body temperature following MRI. The median paired temperature difference was −1.15°C (IQR −1.475 to −0.775). This decrease was statistically significant (97.87% confidence interval −1.5 to −0.7°C, *p* < 0.0001).

To our knowledge this study provides the first documented clinical cohort of febrile children undergoing MRI under sedation or general anesthesia. Despite theoretical concerns that RF energy deposition could exacerbate fever, no patient exhibited temperature elevation. Instead, body temperature decreased consistently in every case.

These findings align with established physiology. While RF‐induced heating has been demonstrated in modeling studies and select clinical settings [3, 4], anesthesia‐related thermoregulatory impairment appears to dominate the thermal response during MRI. Redistribution hypothermia, reduced metabolic heat production, and environmental heat loss outweigh RF heating even in children who begin imaging with significant fever [5, 6, 7].

This cohort therefore adds important empirical evidence to an area previously guided largely by theoretical considerations. Many institutions restrict MRI in febrile children based on theoretical risk rather than empirical evidence. Such policies may delay diagnosis in time‐critical conditions, including deep neck infections, infectious intracranial complications, or febrile status epilepticus. Our data challenge this paradigm, suggesting that MRI does not exacerbate fever and may instead be associated with cooling. While vigilant temperature monitoring remains essential, our findings support reconsideration of strict temperature‐based MRI exclusion criteria when imaging is indicated.

Several limitations warrant acknowledgment. The sample size is small, reflecting the rarity of MRI performed in febrile children, and limits generalizability. Timing of temperature measurements varied; antipyretic administration may have influenced results, and all scans were performed at 1.5 Tesla, potentially limiting extrapolation to 3 Tesla systems. The retrospective, single‐center design further constrains inference.

In conclusion, in this cohort of febrile pediatric patients undergoing MRI, no instances of temperature elevation were observed. Instead, all children experienced a decrease in body temperature. These findings suggest that anesthesia‐related cooling predominates over RF‐associated heating even in febrile children and highlight the need for larger prospective studies to inform evidence‐based guidelines.

## Conflicts of Interest

The authors declare no conflicts of interest.

## Data Availability

The data that support the findings of this study are available from the corresponding author upon reasonable request.
